# The cardiac glycoside ouabain activates NLRP3 inflammasomes and promotes cardiac inflammation and dysfunction

**DOI:** 10.1371/journal.pone.0176676

**Published:** 2017-05-11

**Authors:** Motoi Kobayashi, Fumitake Usui-Kawanishi, Tadayoshi Karasawa, Hiroaki Kimura, Sachiko Watanabe, Nathan Mise, Fujio Kayama, Tadashi Kasahara, Naoyuki Hasebe, Masafumi Takahashi

**Affiliations:** 1 Division of Inflammation Research, Center for Molecular Medicine, Jichi Medical University, Tochigi, Japan; 2 Department of Environmental and Preventive Medicine, Jichi Medical University, Tochigi, Japan; 3 Department of Medicine, Division of Cardiovascular, Respiratory and Neurology, Asahikawa Medical University, Hokkaido, Japan; Toho Daigaku, JAPAN

## Abstract

Cardiac glycosides such as digoxin are Na^+^/K^+^-ATPase inhibitors that are widely used for the treatment of chronic heart failure and cardiac arrhythmias; however, recent epidemiological studies have suggested a relationship between digoxin treatment and increased mortality. We previously showed that nucleotide-binding oligomerization domain-like receptor family pyrin domain-containing 3 (NLRP3) inflammasomes, which regulate caspase-1-dependent interleukin (IL)-1β release, mediate the sterile cardiovascular inflammation. Because the Na^+^/K^+^–ATPase is involved in inflammatory responses, we investigated the role of NLRP3 inflammasomes in the pathophysiology of cardiac glycoside-induced cardiac inflammation and dysfunction. The cardiac glycoside ouabain induced cardiac dysfunction and injury in wild-type mice primed with a low dose of lipopolysaccharide (LPS), although no cardiac dysfunction was observed in mice treated with either ouabain or LPS alone. Ouabain also induced cardiac inflammatory responses, such as macrophage infiltration and IL-1β release, when mice were primed with LPS. These cardiac manifestations were all significantly attenuated in mice deficient in IL-1β. Furthermore, deficiency of NLRP3 inflammasome components, NLRP3 and caspase-1, also attenuated ouabain-induced cardiac dysfunction and inflammation. *In vitro* experiments revealed that ouabain induced NLRP3 inflammasome activation as well as subsequent IL-1β release from macrophages, and this activation was mediated by K^+^ efflux. Our findings demonstrate that cardiac glycosides promote cardiac inflammation and dysfunction through NLRP3 inflammasomes and provide new insights into the mechanisms underlying the adverse effects of cardiac glycosides.

## Introduction

Cardiac glycosides such as digoxin are widely used for the treatment of chronic heart failure and cardiac arrhythmias because of their positive inotropic and negative chronotropic effects [1]; however, there is a growing controversy regarding the relationship between digoxin treatment and mortality. Although earlier studies showed that digoxin treatment improved symptoms and decreased hospitalization rates, without an increased risk of mortality, in patients with heart failure [2], recent epidemiological studies have suggested that digoxin treatment is associated with an increased risk of mortality in patients with atrial fibrillation (AF) [3, 4]. Cardiac glycosides are steroid derivatives that bind to the α-subunit of the sarcolemmal Na^+^/K^+^-ATPase pump and block its activity, increasing intracellular Na^+^ concentration and leading to an increase in intracellular Ca^2+^ concentrations via a Na^+^/Ca^2+^ exchange [1]. This increase in intracellular Ca^2+^ concentration causes positive inotropy in cardiomyocytes. Cardiac glycosides also evoke vagal activation, resulting in a shift in autonomic balance toward parasympathetic predominance and a negative chronotropic effect. Recent investigations have suggested that in addition to its iron-pumping function, the Na^+^/K^+^-ATPase serves as a scaffold protein that interacts with neighboring proteins and potentiates multiple signaling pathways, leading to the activation of transcriptional factors, such as nuclear factor (NF)-κB and activator protein-1. These effects suggest that the Na^+^/K^+^-ATPase modulates inflammatory responses [5–7], but a causal link between the Na^+^/K^+^-ATPase and inflammation is not fully understood.

Recent evidence indicates that some types of sterile inflammation are mediated by nucleotide-binding oligomerization domain-like receptor family pyrin domain-containing 3 (NLRP3) inflammasomes, which are intracellular, multiprotein complexes that regulate the release of the proinflammatory cytokine interleukin (IL)-1β [8, 9]. NLRP3 inflammasomes comprise NLRP3, an apoptosis-associated speck-like protein containing a caspase recruitment domain (ASC), and caspase-1 (Casp1), and serve as a platform for the activation of Casp1. Because Casp1 was previously known as an IL-1β-converting enzyme, its activation induces the processing of pro-IL-1β into its mature form and IL-1β release, leading to inflammatory responses and tissue damage. We have recently demonstrated the involvement of NLRP3 inflammasomes in the pathophysiology of sterile cardiovascular and renal diseases such as myocardial ischemia–reperfusion, atherosclerosis, vascular injury, abdominal aortic aneurysm, chronic kidney disease, and acute kidney injury [10–15]. Furthermore, we and other investigators showed that K^+^ efflux is a key upstream event in the activation of NLRP3 inflammasomes [10, 13]. Because the Na^+^/K^+^-ATPase modulates intracellular K^+^ concentrations [1, 6], we hypothesize that cardiac glycosides could influence NLRP3 inflammasome activation and subsequent IL-1β release in the heart. To test this hypothesis, we used mice deficient in inflammasome-related molecules such as NLRP3, Casp1, and IL-1β, and found that the cardiac glycoside ouabain induces cardiac inflammation and dysfunction via NLRP3 inflammasomes when mice were primed with lipopolysaccharide (LPS). Furthermore, we showed that ouabain induced K^+^ efflux and the subsequent activation of NLRP3 inflammasomes in macrophages. The findings of this study demonstrate a novel role of NLRP3 inflammasomes in the heart and provide new insights into the mechanisms underlying the adverse effects of cardiac glycosides.

## Materials and methods

### Animal protocol

All experiments in this study were approved by the Use and Care of Experimental Animals Committee of the Jichi Medical University Guide for Laboratory Animals (permit number 15082) and were conducted in accordance with the Jichi Medical University guidelines. IL-1β^−/−^, NLRP3^−/−^, and Casp1^−/−^ mice (C57BL/6J genetic background) were kindly provided by Dr. Yoichiro Iwakura (Tokyo University of Science, Chiba, Japan), Dr. Hiroko Tsutui (Hyogo College of Medicine, Nishinomiya, Japan), and Dr. Vishava M. Dixit (Genentech, South San Francisco, CA), respectively [16–19]. Wild-type (WT, C57BL/6J background) mice were purchased from Japan SLC, Inc. (Tokyo, Japan). Mice were housed (4/cage, RAIR HD ventilated Micro-Isolator Animal Housing Systems, Lab Products, Seaford, DE) in an environment maintained at 23 ± 2°C with free access to food and water under a 12-h light and dark cycle with lights on from 7:00 to 19:00. For animal experiments, mice (male, 8–12-week old) were primed with an intraperitoneal injection of a low dose of LPS (0111: B4, Wako Chemicals, 2 mg/kg) and then intraperitoneally administered ouabain (Sigma, 3 mg/kg) 12 h after LPS or saline administration. The same volume of saline was used as the vehicle control. Total 50 mice were used for *in vivo* and *in vitro* experiments (WT, IL-1β^−/−^, NLRP3^−/−^, and Casp1^−/−^ mice: n = 21, 8, 15, 6, respectively). All efforts were made to minimize animal discomfort and suffering.

### Echocardiography

Transthoracic echocardiography was performed using a digital ultrasound system (Vevo2100 imaging System, Visual Sonics, Toronto, Canada) with a 30-MHz probe, as previously described [20]. In brief, after the induction of anesthesia by inhalation of 1.5% isoflurane, two-dimensional targeted M-mode echocardiograms were obtained along the short axis of the left ventricle (LV) at the level of the papillary muscles, and at least three consecutive beats were evaluated. The LV end-diastolic diameter (LVEDD) and LV end-systolic diameter (LVESD) were measured, and the percentage of fractional shortening (%FS) and ejection fraction (EF) were calculated using the standard formula.

### Measurements for creatine phosphokinase (CPK) and IL-1β

The levels of CPK and IL-1β were assessed using the chemical analyzer Fuji-dry-chem (Fuji film Co., Tokyo, Japan) and mouse enzyme-linked immunosorbent assay (ELISA, R&D Systems), respectively, according to the manufacturer’s instructions.

### Histology and immunohistochemistry

Hearts were embedded in Tissue-Tek O.C.T. compound (Sakura Finetechnical Co. Ltd., Tokyo, Japan), and were cut into 4-μm-thick sections, and fixed with Mildform 10N (Wako Pure Chemical Industries, Ltd., Osaka, Japan). The sections were stained with hematoxylin and eosin. Immunohistochemical analysis was performed to detect the pan-leukocyte marker CD45 (BD Bioscience) and the macrophage marker CD68 (Santa Cruz Biotechnology), as previously described [20]. The stained sections were photographed by using a fluorescence microscope (FSX-100; Olympus, Tokyo, Japan) or confocal laser scanning microscope (FV-10i; Olympus). For quantification, the number of CD45^+^ and CD68^+^ cells was counted in 10 randomly chosen fields at a magnification of 200× for each sample.

### Real-time RT-PCR analysis

Total RNA was prepared from the heart tissues using ISOGEN (Nippon Gene Co., Ltd., Toyama, Japan), according to the manufacturer’s instructions. Real-time RT-PCR analysis was performed using the Takara TP960 PCR Thermal Cycler Dice Detection System (Takara Bio Inc., Shiga, Japan) for the measurements of mRNA expression. The primers were as follows: *Il1b*, 5′-TGAAGTTGACGGACCCCAAA-3′ and 5′-TGATGTGCTGCTGTGAGATT-3′; *Nlrp3*, 5′-CGAGACCTCTGGGAAAAAGCT-3′ and 5′-GCATACCATAGAGGAATGTGATGTACA-3′; and *Gapdh*, 5′-TGTGTCCGTCGTGGATCTGA-3′ and 5′-TTGCTGTTGAAGTCGCAGGAG-3′. Gene expression was normalized to *Gapdh* expression using the software provided with the system.

### Western blot analysis

The processing of pro-IL-1β into its mature form was analyzed by western blotting as previously described [13]. Primary antibodies against IL-1β (R&D Systems) and β-actin (Sigma) were used. The expression level of β-actin served as an internal control for protein loading.

### Cytotoxicity assay

Cytotoxicity was determined by measurements of lactate dehydrogenase (LDH) activity using cytotoxicity detection kit (Roche, Mannheim, Germany).

### Cell culture

Murine J774 macrophages were obtained from the RIKEN Gene Bank (Tsukuba, Japan) and cultured in Dulbecco’s modified Eagle’s medium (Wako Chemicals) supplemented with 10% fetal calf serum. Murine peritoneal macrophages were isolated from mice using the thioglycollate elicitation method and then resuspended in RPMI 1640. Z-Tyr-Val-Ala-Asp-fluoromethylketone (YVAD-FMK) was obtained from MBL (Nagoya, Japan). All reagents were obtained from Sigma unless otherwise specified.

### Intracellular K^+^ concentrations

After priming with LPS (100 ng/mL) for 16 h, murine J774 cells were treated with either ouabain (100 μM) for 3 h or nigericin (2.5 μM) for 4 h. The cells were washed with saline and lysed in 10% nitric acid. Intracellular K^+^ concentrations were measured by a polarized Zeeman atomic absorption spectrophotometer (Hitachi Z-5010, Japan).

### Statistical analysis

Data were expressed as the mean ± standard error of the mean (SEM). An unpaired *t*-test was used to compare two groups. For comparisons between multiple groups, the significance of differences in between-group means was determined by a one-way analysis of variance followed by the Tukey–Kramer test. All analyses were performed using SPSS software, version 21 (IBM Japan Ltd., Tokyo, Japan). A *P*-value less than 0.05 was considered statistically significant.

## Results

### IL-1β deficiency attenuates ouabain-induced cardiac dysfunction and injury

**Fig 1:**
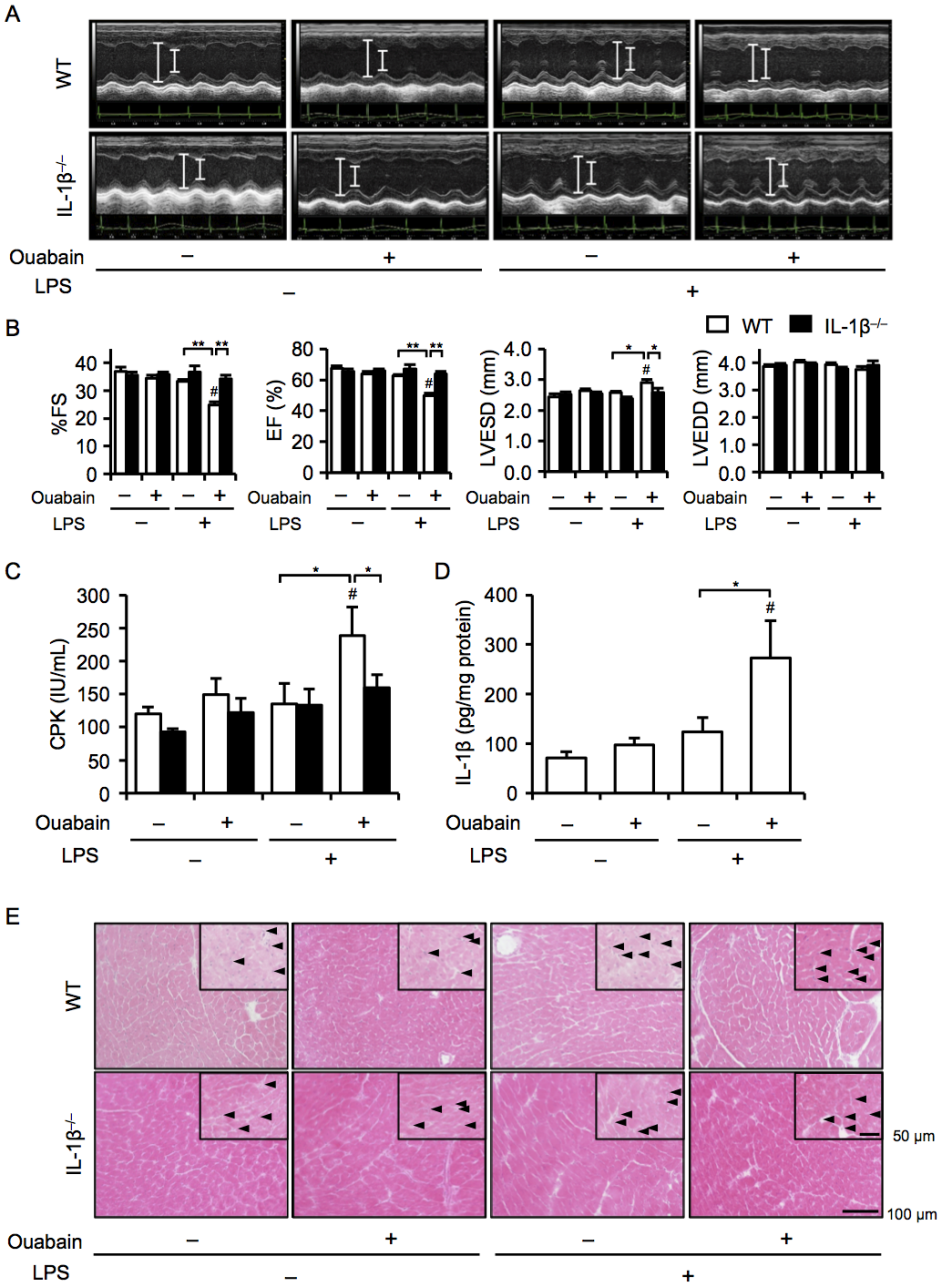
IL-1β deficiency attenuates ouabain-induced cardiac dysfunction and injury. WT and IL-1β^−/−^ mice were treated with ouabain (2 mg/kg) 12 h after LPS (3 mg/kg) administration. Echocardiography was performed, and mice were sacrificed 12 h after ouabain treatment. (A) Two-dimensional M-mode echocardiograms are shown. (B) Cardiac function [%FS and EF (%)] and dimension [LVDs (mm) and LVEDs (mm)] were assessed (n = 8–13 for WT mice, n = 4–7 for NLRP3^−/−^ mice). (C and D) Plasma CPK and cardiac IL-1β protein levels were assessed (n = 4–8 for each). (E) The heart sections were stained with hematoxylin and eosin. Arrowheads indicate infiltrated inflammatory cells. Data are expressed as the mean ± SEM. *P < 0.05 and **P < 0.01. ^#^*P* < 0.05 vs. vehicle (WT).

Because NLRP3 inflammasome-driven IL-1β release requires pro-IL-1β synthesis as a priming signal [9], we examined the dose-dependent effects of ouabain and LPS alone on cardiac dysfunction. We determined 3 mg/kg ouabain and 2 mg/kg LPS to be the subclinical doses. Although treatment with each dose of ouabain or LPS alone showed no cardiac dysfunction in WT mice, ouabain significantly decreased cardiac contractile force with an enlarged LVESD when mice were primed with LPS (Fig 1A and 1B). Consistently, plasma levels of CPK, a marker of cardiac injury, and cardiac tissue IL-1β levels were also elevated in ouabain-treated mice primed with LPS (Fig 1B and 1D). To explore the role of IL-1β in ouabain-induced changes in the cardiac phenotype, we used IL-1β^−/−^ mice and found that cardiac dysfunction and increased CPK levels in response to ouabain were almost completely prevented in IL-1β^−/−^ mice (Fig 1A–1C). A histological examination showed that infiltration of inflammatory cells into the hearts of WT mice treated with ouabain tended to increase, whereas such cellular infiltration was not seen in IL-1β^−/−^ mice (Fig 1E). These findings suggest that IL-1β is essential for ouabain-induced cardiac dysfunction and injury.

### IL-1β deficiency decreases ouabain-induced macrophage infiltration

**Fig 2:**
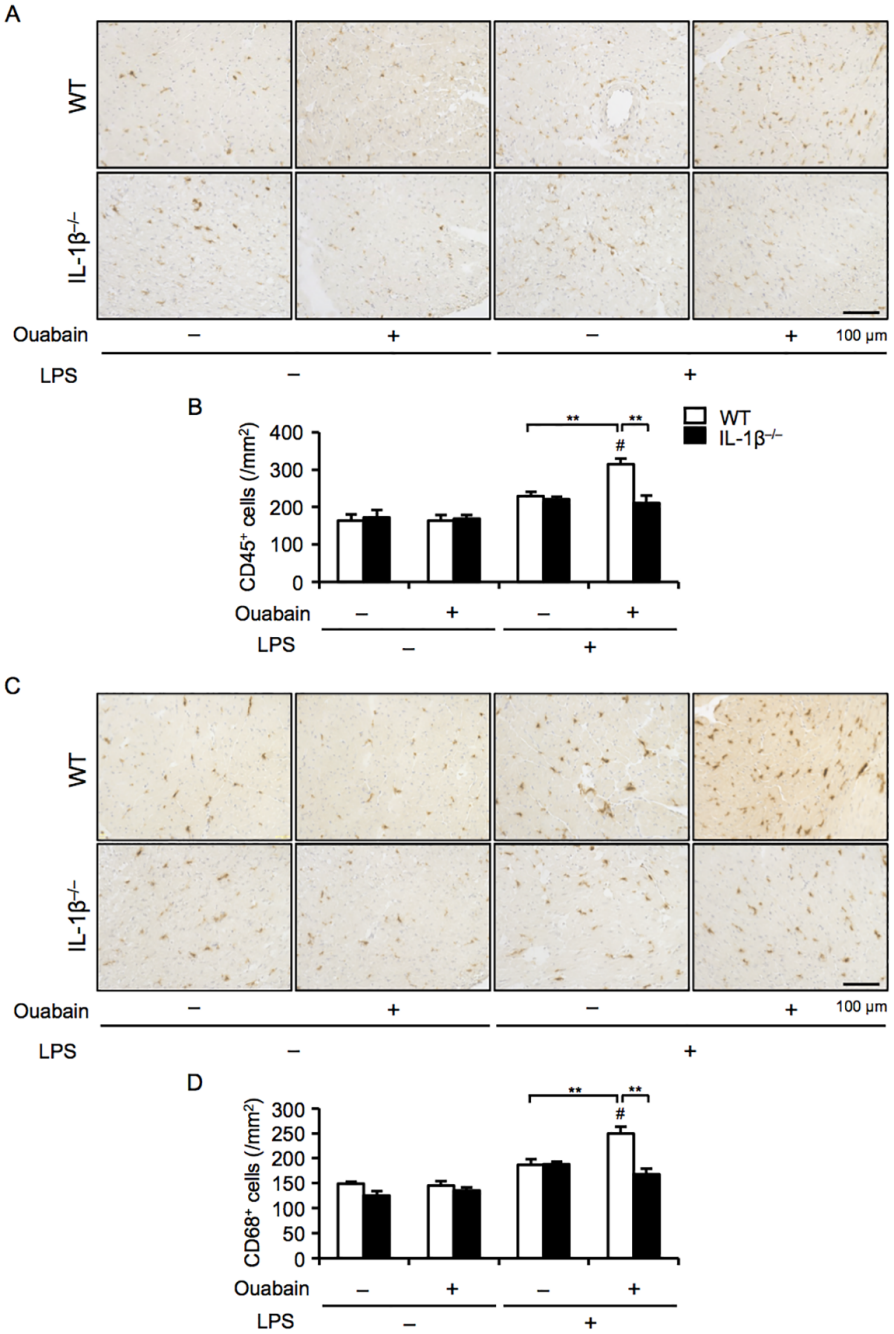
IL-1β deficiency decreases ouabain-induced macrophage infiltration. WT and IL-1β^−/−^ mice were treated with ouabain (2 mg/kg) 12 h after LPS (3 mg/kg) administration and then sacrificed 12 h after ouabain treatment. (A and C) Heart sections were immunohistochemically stained for CD45 and CD68. (B and D) The number of CD45^+^ (leukocytes) and CD68^+^ (macrophages) cells was quantified (n = 4 for each). Data are expressed as the mean ± SEM. *P < 0.05 and **P < 0.01. ^#^*p* < 0.05 vs. vehicle (WT).

Because leukocytes, especially macrophages, are the main cellular source of IL-1β [12], we next examined the infiltration of leukocytes and macrophages into the heart. Immunohistochemical analysis for the pan-leukocyte marker CD45 and the macrophage marker CD68 showed that ouabain significantly increased the number of leukocytes and macrophages that infiltrated in the primed hearts, compared to either LPS priming or ouabain alone (Fig 2A–2D). Furthermore, the infiltration of these cells into the hearts of IL-1β^−/−^ mice was completely inhibited. These findings suggest that IL-1β secreted by infiltrated macrophages contributes to ouabain-induced cardiac inflammation.

### Deficiency of NLRP3 and Casp1 attenuates ouabain-induced cardiac dysfunction and macrophage infiltration

**Fig 3:**
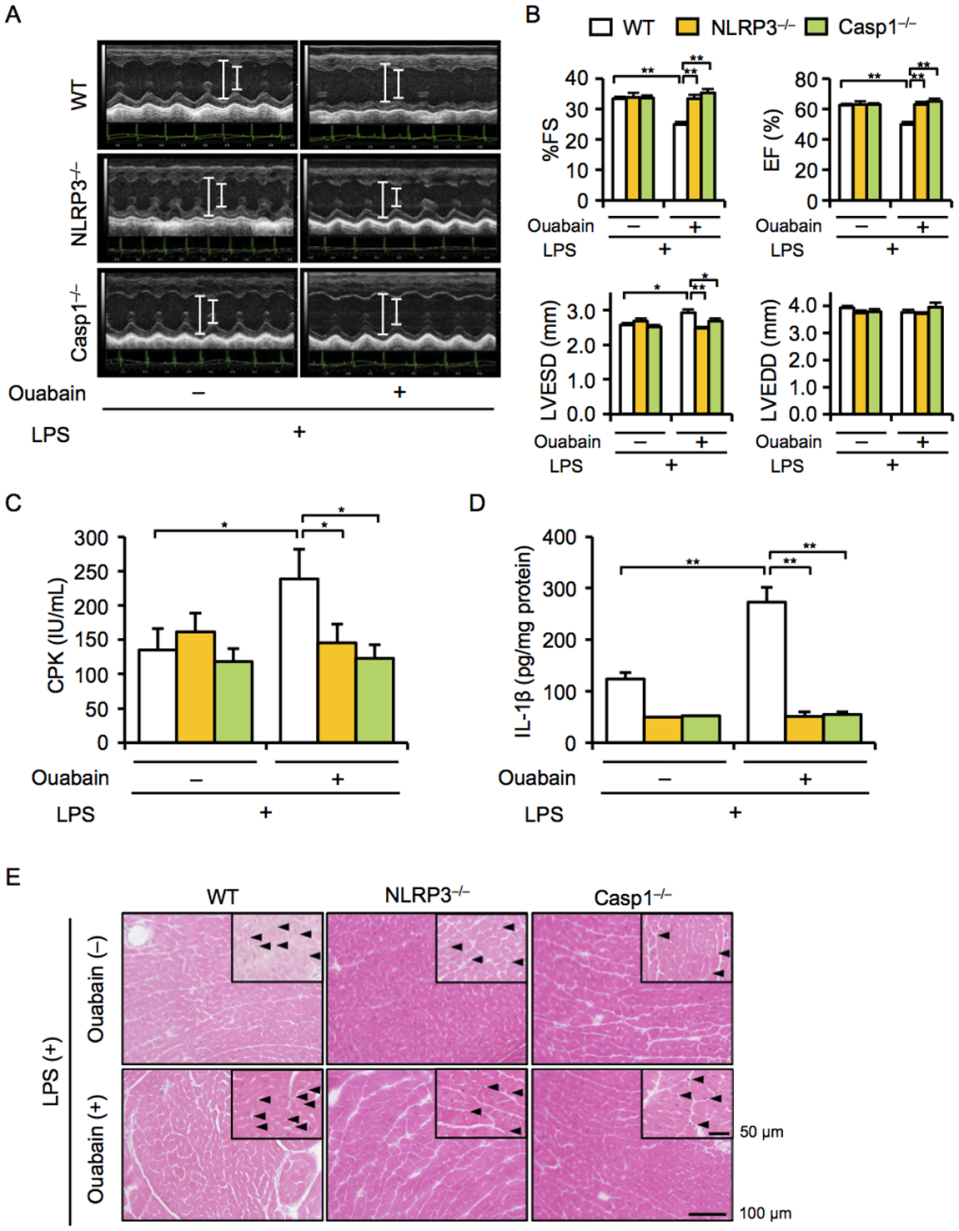
Deficiency in NLRP3 and Casp1 attenuates ouabain-induced cardiac dysfunction. WT, NLRP3^−/−^, and Casp1^−/−^ mice were treated with ouabain (2 mg/kg) 12 h after LPS (3 mg/kg) administration. Echocardiography was performed, and mice were sacrificed 12 h after ouabain treatment. (A) Two-dimensional M-mode echocardiograms are shown. (B) Cardiac function [%FS and EF (%)] and dimension [LVDs (mm) and LVEDs (mm)] were assessed [n = 13 (WT), 8 (NLRP3^−/−^), 6 (Casp1^−/−^)]. (C and D) Plasma CPK and cardiac IL-1β protein levels were assessed [n = 8 (WT), 6 (NLRP3^−/−^), and 5 (Casp1^−/−^)]. (E) Heart sections were stained with hematoxylin and eosin. Arrowheads indicate infiltrated inflammatory cells. Data are expressed as the mean ± SEM. *P < 0.05 and **P < 0.01.

**Fig 4:**
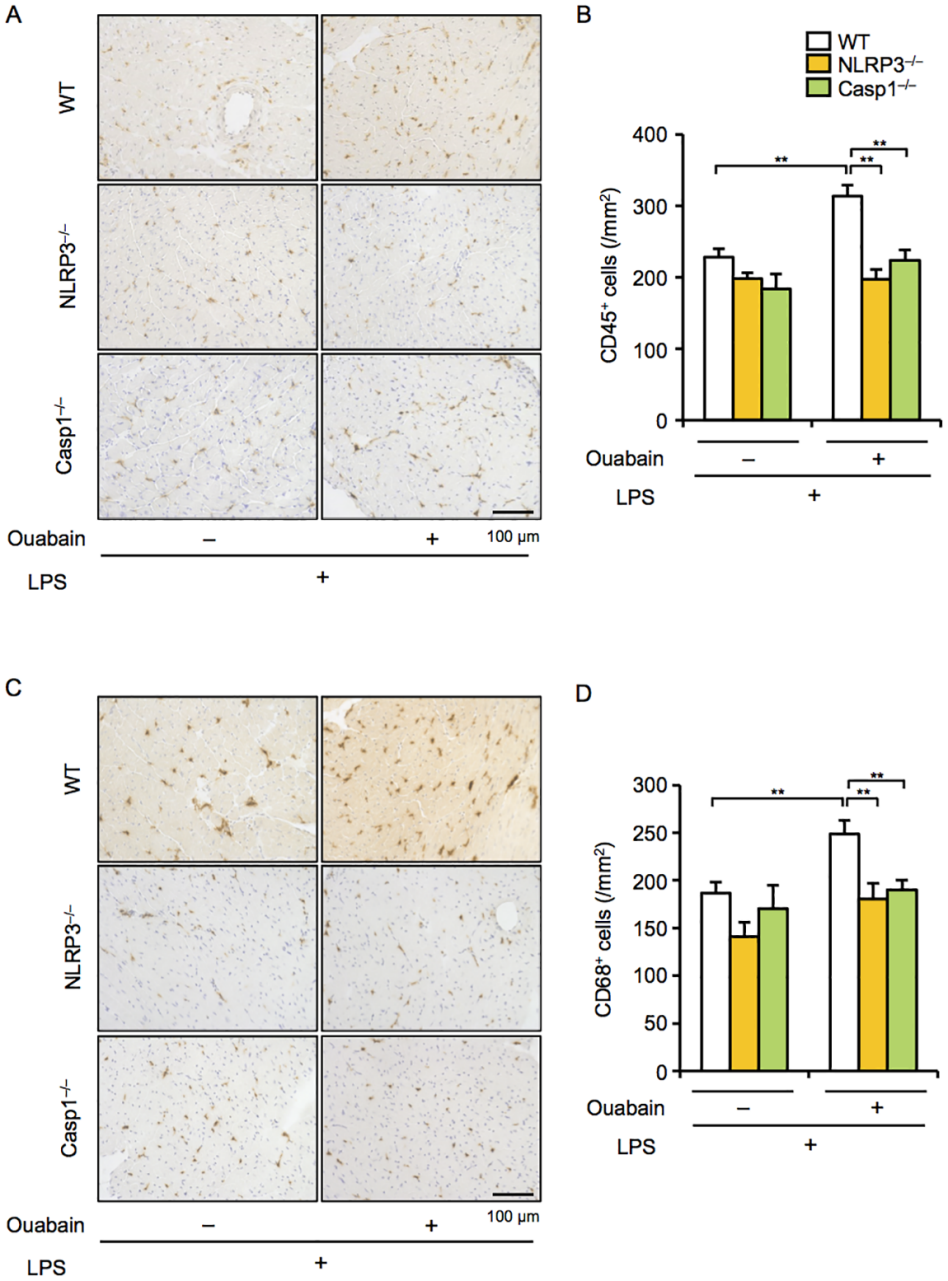
Deficiency in NLRP3 and Casp1 decreases ouabain-induced macrophage infiltration. WT, NLRP3^−/−^, and Casp1^−/−^ mice were treated with ouabain (2 mg/kg) 12 h after LPS (3 mg/kg) administration, and then sacrificed 12 h after ouabain treatment. (A and C) Heart sections were immunohistochemically stained for CD45 and CD68. (B and D) The number of CD45^+^ (leukocytes) and CD68^+^ (macrophages) cells was quantified (n = 4 for each). Data are expressed as the mean ± SEM. *P < 0.05 and **P < 0.01.

IL-1β release from macrophages is regulated by the NLRP3 inflammasomes [12]. To clarify the role of NLRP3 inflammasomes in ouabain-induced cardiac inflammation and dysfunction, we used NLRP3^−/−^ and Casp1^−/−^ mice. Consistent with the findings obtained in IL-1β^−/−^ mice, NLRP3^−/−^ and Casp1^−/−^ mice exhibited significantly attenuated ouabain-induced cardiac phenotypes, including cardiac dysfunction with enlarged LVESD and increased levels of plasma CPK and cardiac IL-1β (Fig 3A–3D). A histological examination also showed that inflammatory cell infiltration into the hearts of NLRP3^−/−^ and Casp1^−/−^ mice tended to be lower than that of WT mice (Fig 3E). Furthermore, the infiltration of CD45^+^ leukocytes and CD68^+^ macrophages into the hearts of NLRP3^−/−^ or Casp1^−/−^ mice was significantly decreased compared to that into the hearts of WT mice (Fig 4A–4D). These results suggest that NLRP3 inflammasomes mediate ouabain-induced cardiac inflammation and dysfunction.

### Ouabain induces NLRP3 inflammasome activation and IL-1β release in macrophages

**Fig 5:**
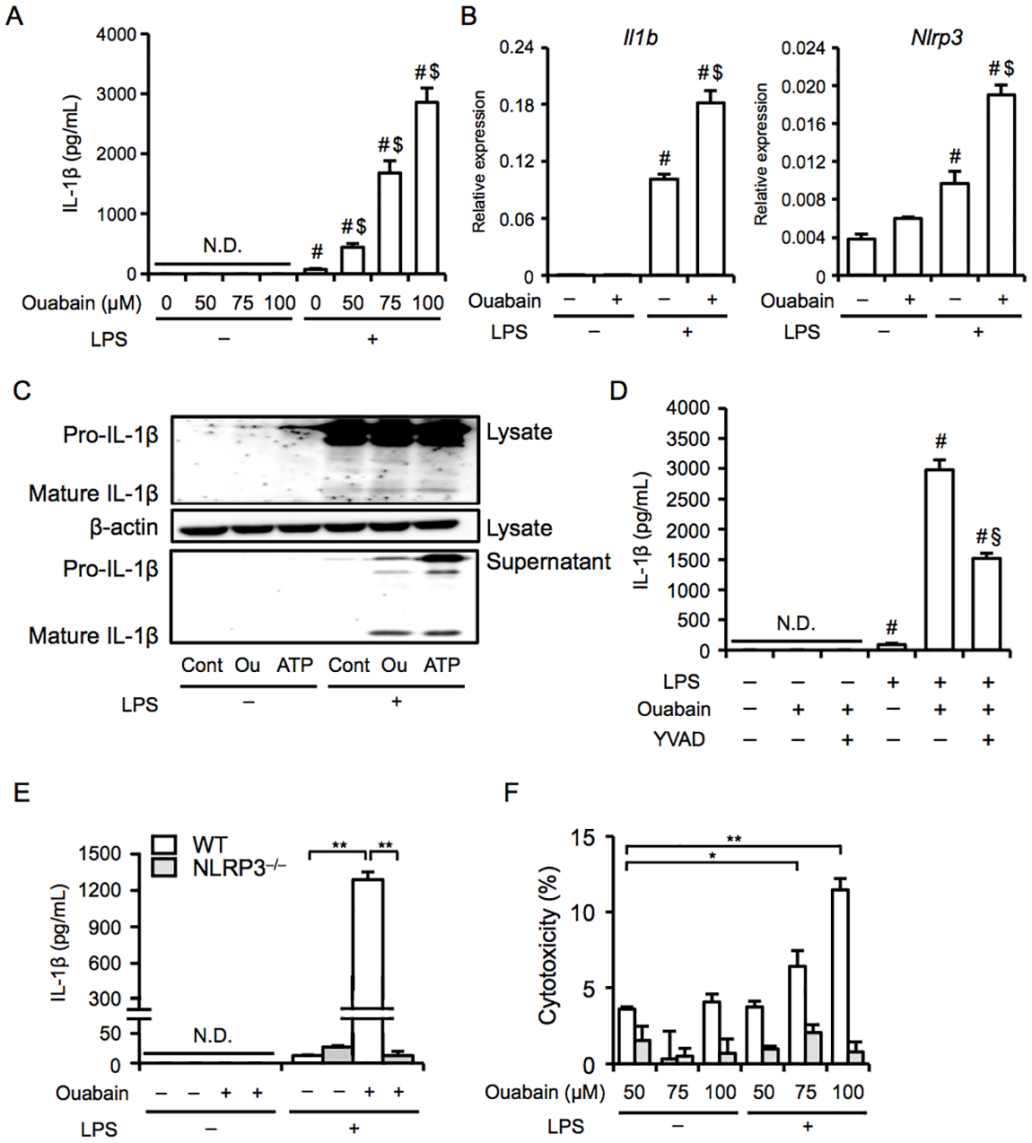
Ouabain induces NLRP3 inflammasome activation and IL-1β release in macrophages. (A–C) After priming with or without LPS (100 ng/mL) for 6 h, J774 macrophages were treated with ouabain (A, 50–100 μM; B and C, 100 μM) for 3 h. (A) IL-1β levels in the supernatants were assessed (n = 6 for each). (B) Heart *Il1b* and *Nlrp3* mRNA levels were assessed by real-time RT-PCR analysis (n = 4 for each). (C) The processing of pro-IL-1β in the lysates and supernatants was assessed by western blot analysis. ATP was used as a positive control. (D) LPS-primed J774 macrophages were treated with ouabain (100 μM) for 3 h in the presence or absence of YVAD-FMK (20 μM). IL-1β levels in the supernatants were assessed (n = 4 for each). (E and F) After priming with or without LPS (100 ng/mL) for 6 h, primary murine macrophages from WT and NLRP3^−/−^ mice were treated with ouabain (E, 100 μM; F, 50–100 μM) for 3 h. (E) IL-1β levels in the supernatants were assessed (n = 4 for each). (F) Cell death was assessed by LDH activity in the supernatants (n = 4 for each). Data are expressed as the mean ± SEM. *P < 0.05 and **p < 0.01. ^#^*P* < 0.05 vs. vehicle. ^$^*P* < 0.05 vs. LPS alone and ^§^*P* < 0.05 vs. Ouabain with LPS.

To investigate the molecular mechanisms by which ouabain promotes NLRP3 inflammasome activation, we used murine macrophage J774 cells and primary peritoneal macrophages. To ensure pro-IL-1 induction, we primed the cells with a low dose of LPS, as previously described [10, 12, 13], and examined the effects of ouabain on IL-1β release. Ouabain induced IL-1β release in primed J774 macrophages in a dose-dependent manner (Fig 5A). As expected, priming with LPS significantly upregulated mRNA expression levels of *Il1b* and *Nlrp3*, which are known as priming signals for NLRP3 inflammasomes [9]. Intriguingly, ouabain further increased the expression levels of *Il1b* and *Nlrp3*, although no induction was detected in response to ouabain alone (Fig 5B). To confirm the processing of pro-IL-1β, we performed western blot analysis and showed that pro-IL-1β was processed into its mature form (17 kDa) by ouabain in both cell supernatants and lysates (Fig 5C). Similar results were obtained when ATP was used as a positive control. In addition, IL-1β release was significantly blocked by the caspase-1 inhibitor YVAD-FMK (Fig 5D). Consistent with the data on J774 macrophages, ouabain markedly stimulated IL-1β release by primary macrophages from WT mice (Fig 5E). This IL-1β release was completely inhibited by macrophages from NLRP3^−/−^ mice. Because NLRP3 inflammasomes have also been shown to induce pyroptosis, i.e., inflammatory cell death accompanied by increased plasma membrane permeability [8, 9], we determined membrane integrity by an LDH release assay. Ouabain induced cell death in a dose-dependent manner in WT macrophages but not in NLRP3^−/−^ macrophages (Fig 5F). These findings suggest that ouabain induces NLRP3 inflammasome activation, leading to subsequent IL-1β release from and inflammatory cell death in macrophages.

### Ouabain-induced NLRP3 inflammasome activation is mediated through K^+^ efflux

**Fig 6:**
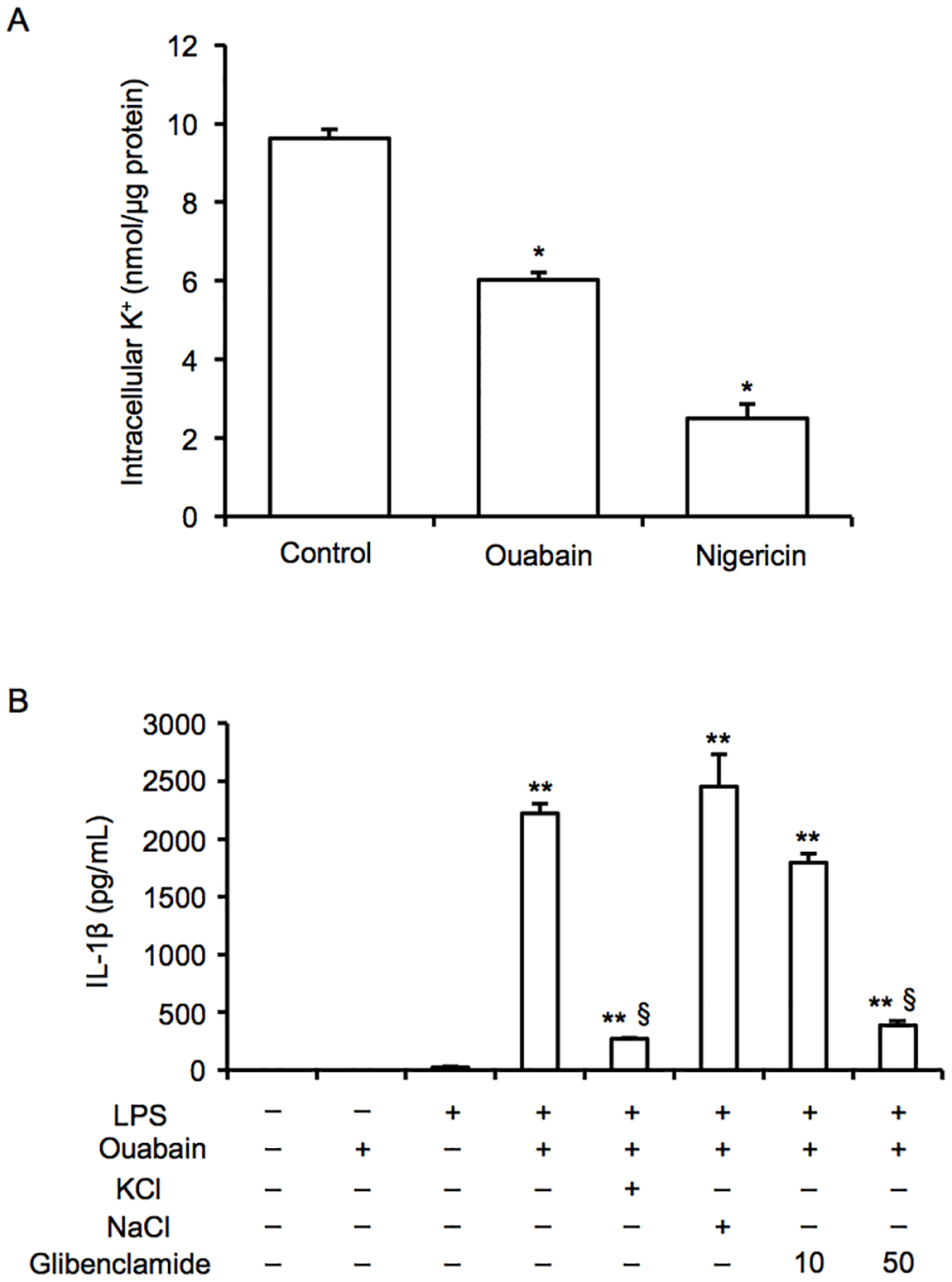
Ouabain-induced NLRP3 inflammasome activation is mediated through K^+^ efflux. (A) After priming with or without LPS (100 ng/mL) for 6 h, J774 macrophages were treated with ouabain (100 μM) for 3 h or nigericin (3.4 μM) for 2 h. The cells were lysed and intracellular K^+^ concentrations were assessed by using an atomic absorption spectrometry (n = 4 for each). (B) LPS-primed J774 macrophages were treated with ouabain (100 μM) for 3 h in the presence or absence of KCl (130 mM), NaCl (130 mM), or glibenclamide (10 and 50 μM). IL-1β levels in the supernatants were assessed (n = 4 for each). Data are expressed as the mean ± SEM. *P < 0.05 and **P < 0.01. ^#^*p* < 0.05 vs. vehicle and ^§^*P* < 0.05 vs. Ouabain with LPS.

It is known that K^+^ efflux is an important trigger of NLRP3 inflammasome activation [8, 9]. In addition, because ouabain inhibits the Na^+^/K^+^-ATPase, which influences intracellular K^+^ concentrations, we tested the role of K^+^ efflux in the ouabain-stimulated activation of NLRP3 inflammasomes in macrophages. Analysis using an atomic absorption spectrometry showed that ouabain clearly decreased intracellular K^+^ concentrations in J774 macrophages (Fig 6A). Nigericin, a K^+^ ionophore, was used as a positive control. To block K^+^ efflux in macrophages, we treated these cells with a high concentration of KCl (130 mM) or the potassium channel blocker, glibenclamide (10–50 μM) and showed that the inhibition of K^+^ efflux significantly prevented ouabain-induced IL-1β release (Fig 6B). In contrast, treatment with a high concentration of NaCl (130 mM) had no effect on ouabain-induced IL-1β release. These findings indicate that ouabain-promoted activation of NLRP3 inflammasomes is mediated through, at least in part, K^+^ efflux.

## Discussion

The major findings of this study are as follows: (1) ouabain induces cardiac dysfunction and injury in WT mice primed with a low dose of LPS; (2) ouabain induces cardiac inflammatory responses, such as macrophage infiltration and IL-1β release; (3) the aforementioned cardiac manifestations are all significantly attenuated in mice deficient in NLRP3 inflammasome-related molecules, such as NLRP3, Casp1, and IL-1β; (4) ouabain induces NLRP3 inflammasome activation and subsequent IL-1β release in macrophages primed with a low dose of LPS; and (5) ouabain-induced activation of NLRP3 inflammasomes is mediated through K^+^ efflux. The results of the present study demonstrate that the Na^+^/K^+^-ATPase inhibitor ouabain induces NLRP3 inflammasome activation through K^+^ efflux, leading to cardiac inflammation and dysfunction. Because recent epidemiologic studies have suggested that digoxin therapy is associated with an increased risk of mortality, particularly in patients with AF [3, 4], our findings demonstrate a novel action of cardiac glycosides and provide new insights into the causal link between the use of cardiac glycosides and their potential adverse effects.

The Na^+^/K^+^-ATPase is a conserved transmembrane protein that maintains the electrochemical gradient using ATP as its energy source; it also functions as a receptor for cardiac glycosides, such as digoxin and ouabain [1, 6, 7]. Cardiac glycosides bind to and inhibit the Na^+^/K^+^-ATPase and have been used in patients with heart failure for more than 200 years. Although digoxin is still widely used to treat heart failure and AF, recent epidemiological studies suggested that digoxin therapy is associated with an increased risk of mortality [3, 4]. However, the mechanism underlying this association remains unknown. Recent evidence indicates that cardiac glycosides can modulate inflammatory processes via the activation of several distinct signaling pathways, including the Src/mitogen-activated protein kinase pathway and the phosphoinositide 3-kinase/Akt pathway [6, 7]. These signals result in the activation of the inflammatory-associated transcription factor NF-κB and the subsequent production of inflammatory cytokines. In contrast, however, several reports showed that ouabain has the ability to suppress the production of inflammatory cytokines, suggesting an anti-inflammatory effect of cardiac glycosides [7]. Thus, the precise role of the Na^+^/K^+^-ATPase in inflammation remains controversial. In the present study, we clearly demonstrate that ouabain induces cardiac inflammation and dysfunction in mice primed with a low dose of LPS, and that these cardiac manifestations are clearly attenuated by deficiency in NLRP3, Casp1, and IL-1β. With respect to the relationship between cardiac glycosides and inflammatory responses in the heart, Zhang *et al*. [21] recently reported that the inhibition of the Na^+^/K^+^-ATPase activates Ca^2+^-dependent mammalian target of rapamycin signaling and subsequently promotes cardiac tumor necrosis factor-α expression in mice treated with LPS. At present, however, no information is available on the role of NLRP3 inflammasomes in ouabain-induced cardiac inflammation. To our knowledge, this study provides the first evidence that NLRP3 inflammasomes contribute to the process of cardiac glycoside-mediated cardiac inflammation.

In the present study, we showed that macrophages are the predominant effector cells that activate NLRP3 inflammasomes and produce IL-1β in the heart; this is based on the following findings. First, deficiency in NLRP3 inflammasomes and IL-1β significantly inhibits the infiltration of macrophages into the heart. Second, NLRP3 inflammasomes are mainly expressed in cells of a myeloid lineage, such as macrophages and dendritic cells [8, 9]. Third, we recently identified that macrophages are the predominant inflammatory cells in the heart [20]. In this regard, we previously showed that inflammasome activation of cardiac fibroblasts plays an essential role in the initial inflammatory response after myocardial ischemia-reperfusion [10]. This initial inflammatory response in the cardiac fibroblasts stimulates the release of cytokines/chemokines, which recruit and activate monocytes/macrophages to the ischemic myocardium. Activation of the infiltrated macrophages by the inflammasome further enhances the inflammatory response and leads to myocardial injury. Moreover, cardiomyocytes also have components of NLRP3 inflammasomes and their activation directly induces cardiomyocyte cell death called “pyroptosis” [22]. Therefore, we assume that cardiac fibroblasts and cardiomyocytes may play a role in ouabain-induced cardiac inflammation.

Another issue to be noted is that the mechanisms underlying cardiac glycoside-induced activation of NLRP3 inflammasomes. We clearly show that ouabain induces K^+^ efflux in macrophages, which mediates the activation of NLRP3 inflammasomes. This finding is understandable because inhibition of the Na^+^/K^+^-ATPase induces intracellular K^+^ depletion. Indeed, we observed that ouabain treatment significantly decreases intracellular K^+^ concentrations in macrophages. In addition to K^+^ efflux, however, several common pathways upstream of NLRP3 inflammasome activation have been identified, including those involving lysosomal destabilization and mitochondrial reactive oxygen species (ROS). Of these, Liu *et al*. [6] reported that ouabain activates the Src/Ras signaling pathway and leads to the generation of mitochondrial ROS in cardiomyocytes. Further investigations are necessary to elucidate the precise mechanism underlying ouabain-induced NLRP3 inflammasome activation in the heart.

In summary, we demonstrate that the inhibition of the Na^+^/K^+^-ATPase by ouabain induces cardiac inflammation and dysfunction via NLRP3 inflammasome-driven IL-1β release. Because cardiac glycosides including ouabain and digoxin inhibit Na^+^/K^+^–ATPase and lead to leads to K^+^ efflux, which is the common trigger of NLRP3 inflammasome activation [8, 9], we assume that other cardiac glycosides can induce NLRP3 inflammasome activation. Our findings provide important information on the mechanism underlying cardiac glycoside-related adverse events. Of note, both ouabain-induced IL-1β release and changes in cardiac phenotype were detected only when mice were primed with an infectious agent (e.g., LPS) to ensure pro-IL-1β induction. Because it is well-known that elderly patients are more susceptible to infections, we must be aware of the potential risk that cardiac glycosides pose in terms of the induction of cardiac adverse effects in these patients with chronic heart failure and cardiac arrhythmias. Our study identifies a novel role of NLRP3 inflammasomes and suggests a potential therapeutic target for cardiac glycoside-related adverse effects.

## Supporting information

S1 Fig The whole membrane by western blot analysis of Fig 5C. (PDF)

S2 Fig NC3Rs ARRIVE guidelines check list. (PDF)
