# Supplementary figures and images for: The cardiac glycoside ouabain activates NLRP3 inflammasomes and promotes cardiac inflammation and dysfunction
Source: PLoS One. 2017 May 11;12(5):e0176676. doi: 10.1371/journal.pone.0176676 (PMC5426608; doi:10.1371/journal.pone.0176676)

Fig 5C, Kobayashi et al.

Lysate IL-1 $\beta$

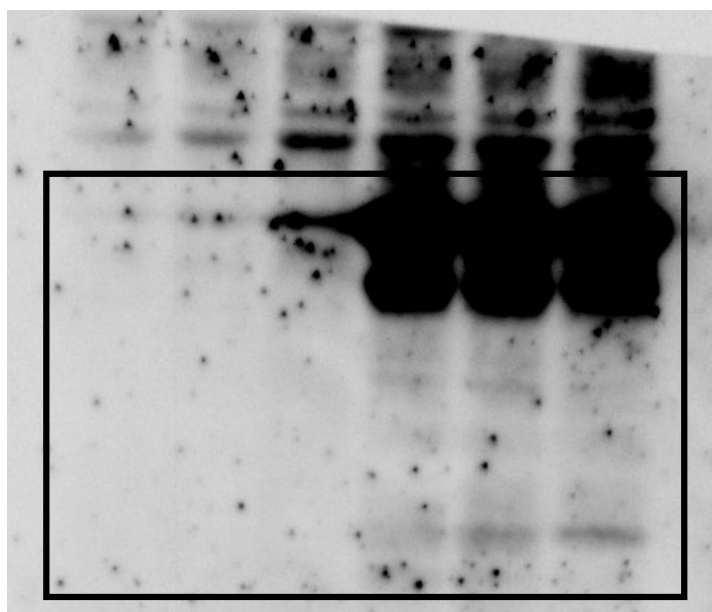

Lysate  $\beta$ -actin

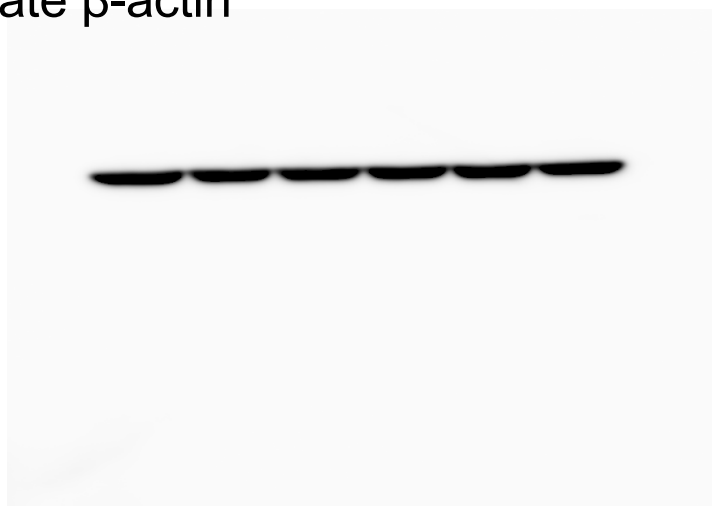

Supernatant IL-1 $\beta$

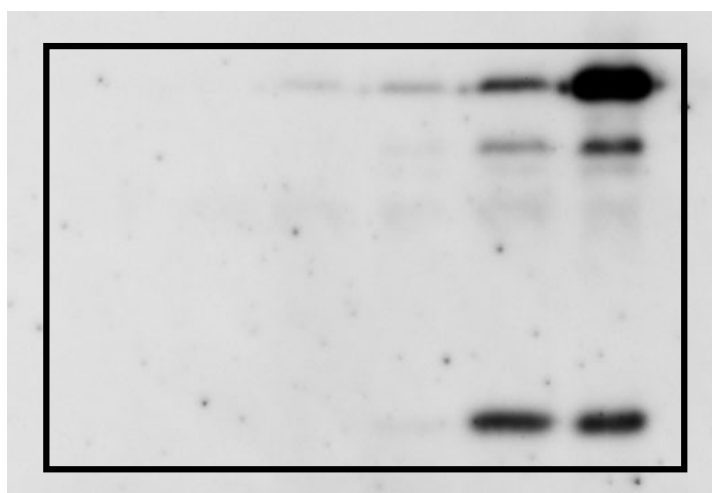

Supplement: S1 Fig — (PDF) [file pone.0176676.s001.pdf]
